# An Effective Scheduling Algorithm for Coverage Control in Underwater Acoustic Sensor Network

**DOI:** 10.3390/s18082512

**Published:** 2018-08-01

**Authors:** Hui Wang, Youming Li, Tingcheng Chang, Shengming Chang

**Affiliations:** 1 Department of Electrical Engineering and Computer Science, Ningbo University, Ningbo 315211, China; wangh0802@163.com (H.W.); csm20130504@163.com (S.C.); 2 Department of Computer Science Engineering, Ningde Normal University, Ningde 352000, China; 18250922163@163.com

**Keywords:** underwater acoustic sensor network, coverage maintenance, memetic algorithm, sleep–wake scheduling

## Abstract

Coverage maintenance is a bottleneck restricting the development of underwater acoustic sensor networks (UASNs). Since the energy of the nodes is limited, the coverage of UASNs may gradually decrease as the network operates. Thus, energy-saving coverage control is crucial for UASNs. To solve the above problems, this paper proposes a coverage-control strategy (referred to as ESACC) that establishes a sleep–wake scheduling mechanism based on the redundancy of deployment nodes. The strategy has two main parts: (1) Node sleep scheduling based on a memetic algorithm. To ensure network monitoring performance, only some nodes are scheduled to work, with redundant nodes in a low-power hibernation state, reducing energy consumption and prolonging the network lifetime. The goal of node scheduling is to find a minimum set of nodes that can cover the monitoring area, and a memetic algorithm can solve this problem. (2) Wake-up scheme. During network operation, sleeping nodes are woken to cover the dead nodes and maintain high coverage. This scheme not only reduces the network energy consumption but takes into account the monitoring coverage of the network. The experimental data show that ESACC performs better than current algorithms, and can improve the network life cycle while ensuring high coverage.

## 1. Introduction

With the rise of the marine economy, countries around the world are paying increased attention to maritime rights and interests. Hence, underwater acoustic sensor networks (UASNs) have become a popular research topic [1,2]. They are mainly applied to mid- and long-term marine-resource surveys, disaster warning and forecasting, environmental monitoring, and submarine exploration [3]. Their broad application prospects have gained attention in academic and military circles.

The sensing range of nodes in UASNs is known to be limited. Therefore, the coverage of a monitored area is an important issue that UASNs must consider in topology control [4,5,6,7]; in addition, UASNs are node-sparse networks, so the coverage problem within the scope of monitoring is more difficult to solve, as the failure of any one node may lead to coverage holes. However, underwater nodes are expensive, and their redundancy is costly and difficult to implement. Reasonable planning is required for coverage and redundancy.

To achieve energy optimization, early researchers experimented with many algorithms and strategies for different network structures and application requirements [8,9,10,11,12]. This research has two main aspects.

The first aspect is to achieve energy balance through network topology control, which is mainly achieved by equalizing energy loss within the entire network or optimizing node transmit power. Based on network-topology control, the energy consumption of the entire network is balanced, so that the lifetime of the wireless sensor network is extended, or the node transmit power-control algorithm is optimized. According to the redundancy of the deployment node, optimization and balance are achieved through the sleep–wake rotation of nodes. Based on different characteristics of nodes, the residual energy aware dynamic algorithm [13] dynamically senses the residual energy at nodes, determines the transmit power based on that, and builds a topology relationship. This is aimed at extending the network lifetime by reducing the use of network nodes with low remaining energy for data forwarding. The algorithm uses a centralized control-based topology construction algorithm. It needs to know the topology information of the entire network, and the overhead is too large. A local topology-control algorithm [14] is proposed to avoid the addition of nodes with low residual energy to the forwarding path. This is a distributed topology control algorithm. Fewer algorithms are based on power control and consider the remaining energy of a node. In underwater sensor network topology control, there is still a lack of simple and effective methods.

The second aspect is to implement energy optimization through routing strategies. Energy optimization is mainly implemented by reducing the routing overhead. Methods of reducing routing overhead include minimum transmission energy consumption routing, maximum network lifetime routing, and path energy consumption equalization policies. The low-energy adaptive clustering hierarchy (LEACH) protocol [15] is a typical clustering algorithm. Through the cluster head selection strategy, the topology relationship is rebuilt on each selection. The geographical adaptive fidelity (GAF) algorithm [16] is a clustering algorithm based on the geographic location of nodes. The algorithm divides the monitoring area into several cells, assigns nodes to cells according to geographical locations, and periodically selects a cluster head node in each cell. The GAF algorithm is based on the plane model, and it uses the distance between nodes to judge whether they can communicate with each other. However, in practical applications, it is not possible to consider only the physical location, such as the communication channel. Moreover, the algorithm does not consider the problem of node energy balance.

Each of the above energy-optimization algorithms focuses on different scenarios and application modes, and some algorithms have good results in the context of their design. At present, most of these achievements are for the design of land-surface wireless sensor networks. Underwater sensor networks have some common problems, as summarized below.

(1) In the research object, the system model does not consider the water environment factors in practical application, and cannot meet the energy-optimization requirements of the underwater sensor network.
(2) The autonomy of network nodes is insufficient, and there is a lack of intelligence in the process of energy-optimization of nodes.

To solve the above problems, this paper proposes an energy-saving coverage-scheduling scheme to optimize UASNs. We studied the node sleep–wake scheduling mechanism based on a memetic algorithm, closed the redundant nodes in the network after node deployment, and optimized the UASN’s topology. It is worth noting that the coverage area of a redundant node usually overlaps with that of its neighbor nodes. Switching a redundant node to sleep mode can reduce network overhead. Further, an effective node wake-up scheme is also a problem considered in this paper. With the operation of the network, the energy of some nodes is depleted, which can easily cause network cavities. The wake-up scheme will activate one or more nodes in an affected neighborhood to maintain high coverage. The ESACC algorithm achieves maximum coverage and extends the entire network life cycle with a minimum number of active nodes. This study theoretically analyzed and evaluated the performance of the algorithm through experiments. The results show that the algorithm is superior to the CMSS [6] and LEACH-Coverage-U  [11] algorithms in maintaining high coverage and a long lifetime.

The rest of this paper is organized as follows: Section 2 describes the preconditions and models related to the ESACC algorithm. Section 3 provides a detailed description of the issues studied in this paper and the proposed ESACC algorithm. Section 4 describes our experimental results and compares them with different methods. Conclusions are given in Section 5.

## 2. Preliminaries

### 2.1. Assumptions

We assume that the monitoring area is a cube denoted by *A*, and the monitoring objects are randomly distributed events and the number of events is G. Assume that the sink node is stationary after deployment (ignoring small-scale movements), and the nodes are not restricted. The sink node and all other nodes can use the relevant positioning algorithm to know their real-time location in the monitored waters. In addition, assume that all nodes are homogeneous and have the same communication capacity, sensing range, and data-processing capability. The sensing range of a node is a spherical area with sensing radius $r_{s}$.

**Figure 1:**
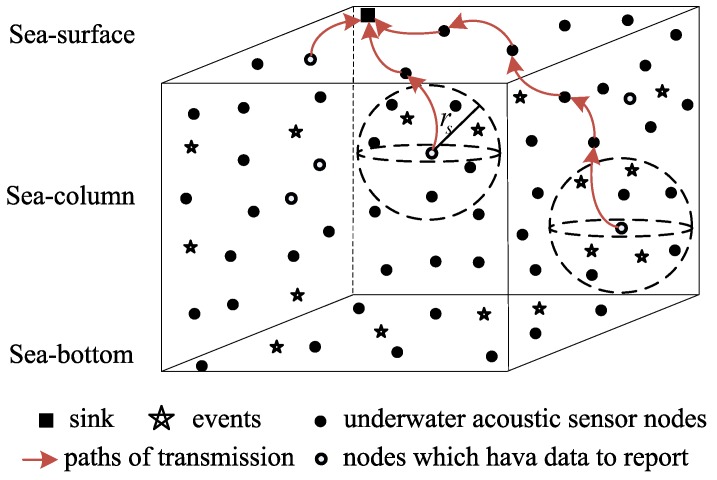
Three-dimensional underwater acoustic sensor network structure.

The method proposed in this paper accounts for the coverage of a given area. Here, we assume that each event $e_{t}$ will always generate an event signal. As shown in Figure 1, each event $e_{t}$ is surrounded by multiple nodes. If $e_{t}$ is within the perceived range of a given node $r_{s}$, the node will detect the event signal and transmit the measured data to the receiver through multi-hop mode.

### 2.2. Sensing Coverage Model

The perceptual coverage model is used to describe the node’s perception of the surrounding waters [17]. Assuming there is a node $s_{i}\left(x_{i},y_{i},z_{i}\right)$ in the three-dimensional space, its Euclidean distance to any spatial event $e_{j}\left(x_{j},y_{j},z_{j}\right)$ is $d(s_{i},e_{j})$. The binary detection model determines that the probability that event $e_{j}$ is covered by node $s_{i}$ is

(1) $$\begin{array}{c} \gamma_{ji}=\left\{\begin{array}{cc} 1 & d\left(s_{i},e_{j}\right)=\sqrt{{\left(x_{i}-x_{j}\right)}^{2}+{\left(y_{i}-y_{j}\right)}^{2}+{\left(z_{i}-z_{j}\right)}^{2}}<r_{s}\\ 0 & otherwise \end{array}\right. \end{array}$$

Equation (1) shows that, in monitoring area *A*, for $\forall e_{j}\in A$, if the Euclidean distance between the event $e_{i}$ and the node $s_{i}$ is smaller than the sensor radius $r_{s}$, then it can be covered, and otherwise it is not covered. Assume that there are *k* nodes $s_{1}$, $s_{2}$, ⋯, $s_{k}$, and their coverage rates for event $e_{i}$ are $\gamma_{i1}$, $\gamma_{i2}$, ⋯, $\gamma_{ik}$ respectively. Then, the coverage of event $e_{i}$ in the network is

(2) $$\begin{array}{c} \gamma_{A}\left(e_{i}\right)=1-\prod_{n=1}^{k}\left(1-\gamma_{in}\right) \end{array}$$

### 2.3. Network Energy-Consumption Model

Much literature has proposed node energy-consumption models for UASNs [18,19]. This paper uses the same energy-consumption model as Zhao and Liang (2006) [19]. The energy consumed by the transmitter to send a package of *m* bits is

(3) $$\begin{array}{c} E_{TX}\left(m,d\right)=mE_{elec}+mT_{b}CHde^{a\left(f\right)d} \end{array}$$

Similarly, the energy consumption of the receiver is

(4) $$\begin{array}{c} E_{RX}\left(m,d\right)=mE_{elec} \end{array}$$

where $E_{elec}$ is the energy consumed by the node circuit to process one bit of information. *C* is the coefficient when the transmitter is at the minimum transmit power derived from the sonar equation in [19], and its value is $2\pi \times 0.67\times 10^{-9.5}$. *H* is the average depth of the node, and *d* is the transmission distance. $T_{b}$ is the duration of one bit, which can be expressed as [20]

(5) $$\begin{array}{c} T_{b}=\frac{M^{\prime }}{S_{v}} \end{array}$$

Here, $M^{\prime }$ is the size of the packet to be sent, $S_{v}$ is the data transmission rate, and a(f) is the absorption coefficient that depend on frequency [18,21]:

(6) $$\begin{array}{c} a\left(f\right)=\frac{0.11f^{2}}{1+f^{2}}+\frac{44f^{2}}{4100+f^{2}}+2.75\times 10^{-4}f^{2}+0.003 \end{array}$$

where *f* is the transmission frequency of the signal in kHz and the absorption coefficient a(f) has the unit dB/m, and it is the frequency correlation coefficient. The determination of Equation (6) can be done based on Thorp’s model [22].

## 3. Energy-Efficient Coverage Control Using Memetic Algorithm

### 3.1. Problem Formulation

Assume that there are *G* monitoring events in the underwater monitoring area *A*, which can all be completely covered by the union of the sensing ranges of the *N* nodes. Then, the problem can be described as follows: after giving a set of nodes $S=\left\{s_{1},s_{2},\cdots ,s_{N}\right\}$ and an event set $E=\left\{e_{1},e_{2},\cdots ,e_{G}\right\}$ that can be covered by *S*, find the smallest subset of sensors $S^{\prime }$ so that *E* can be completely covered by $S^{\prime }$ . That is, if the perceived range of node $s_{i}$ is expressed as $r_{s}$ , and the smallest subset of nodes found is $S^{\prime }=\left\{s_{1},s_{2},\cdots ,s_{m}\right\}$ , 1≤m≤N, then $S^{\prime }$ must satisfy $A\subseteq r_{1}⋃r_{2}⋃\cdots ⋃r_{m}$. This is a set-covering problem (SCP) of NP (Non-deterministic Polynomial) difficulty. In this study, SCP will be applied to node-optimization scheduling to solve the problems of energy efficiency and coverage, which can be described as follows:

(7) $$\begin{array}{c} Minimize:\;\sum_{i\in N}c_{i}\cdot q_{i}\;\;\;\;\; \end{array}$$

(8) $$\begin{array}{cc} & Subject\;to:\;\sum_{i\in N}\gamma_{ji}\cdot q_{i}\geq 1\;j\in G \end{array}$$

where $c_{i}$$\left(i\in N\right)$ is the active cost of the *i*th node, and $q_{i}$ is a key decision variable, which is the focus of this study ($q_{i}$ = 1 indicates the node is active, and $q_{i}$ = 0 means the node is asleep). The objective function in Equation (7) describes the number of least active nodes. We assume that the active costs of each node are the same. For example, $c_{i}$ = 1. The constraint in Equation (8) indicates that each monitoring event $e_{i}$ is covered by at least one node. When the network is in working condition, the optimization model described by Equations (7) and (8) is important, as it greatly improves the network energy efficiency and prolongs the lifetime of the network while guaranteeing coverage. The swarm intelligence optimization algorithm is a heuristic algorithm formed by simulating the laws of nature or biology [23,24]. Similar to a genetic algorithm, particle swarm algorithm, memetic algorithm (MA), or fish school algorithm [25,26,27], it has the characteristics of self-learning, self-organization, and self-adaptation, and it provides new optimization methods for UASNs. Based on this, we developed a swarm intelligence optimization algorithm that simulates the evolutionary mechanism of organisms through iterative computations and explores the optimal solution in the solution space. To solve the SCP problem, we propose an effective scheduling algorithm of coverage control (ESACC) and apply it to the UASN. ESACC includes two optimization strategies: (1) node sleep scheduling based on a memetic algorithm, which can suppress redundant nodes in UASNs; and (2) a node wake-up scheme to solve energy-saving coverage optimization problems in each round. In UASNs, a round is a basic unit of time. The ESACC is described below.

### 3.2. Node Sleep-Scheduling Mechanism Based on Memetic Algorithm

The Memetic algorithm was first proposed by Moscato in 1989, which consists of an individual-based local search and a population-based global search to solve large-scale combinatorial optimization problems [27,28]. The initial state of the population is generated randomly or in a specific way. The evolutionary process is operated by genetic algorithms including selection, crossover, and mutation. In ESACC, each individual represents a solution with a corresponding adaptability, which is given by the adaptive function. The adaptive function is used to evaluate the superiority of the solution. After completing the operation of the genetic algorithm, the local search further improves the superiority of the solution. In this way, a new batch of excellent individuals is generated, and the individuals in the new population are closer to the global optimal solution. Below, we describe the redundant node sleep-scheduling algorithm.

#### 3.2.1. Coding Rule Design

The coding is a mapping from the problem space to the solution space. The genetic algorithm cannot directly process the solution data of the solution space. It must map the variables of the solution space into the data structure of the evolutionary space, i.e., the chromosome, before searching. In the coverage of UASNs, each chromosome represents a node-deployment scenario in which each gene describes the state of the node. In this study, the nodes in the UASNs are activated or asleep through proper scheduling to extend the network life cycle. For scheduling, we use binary 0 and 1 to represent the working state of the node (where 0 means sleep and 1 means a node is active).

**Figure 2:**
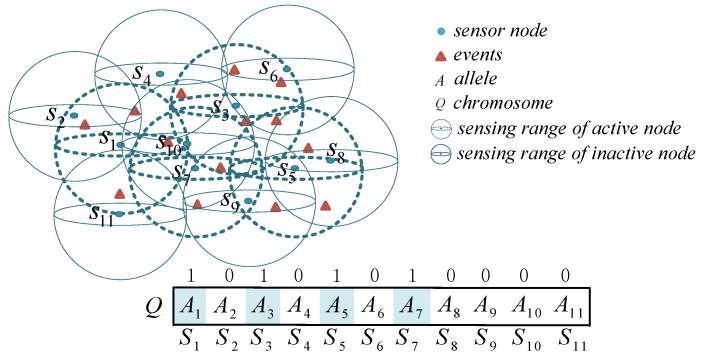
Coding rule design.

As shown in Figure 2, there are 11 nodes that monitor 14 events, and some nodes are redundant (marked with solid lines). To achieve energy-saving optimization coverage, the optimal node-scheduling strategy is integrated into the ESACC so that the redundant nodes go to sleep. From the genetic coding point of view, we use the encoding format “10101010000” to represent the chromosome. N represents the length of the chromosome, whose size equals the number of nodes (N is 11). The gene $A_{j}$ represents the state of node $s_{j}$ in chromosome *Q*. In addition, because diversity during genetic evolution varies with population size, the selection of the population size is critical for ESACC. To simplify this work, based on the opinion of Trivedi et al. [29], the population size of each generation is fixed at 50.

#### 3.2.2. Adaptive Function

An adaptive function is used to evaluate the degree of chromosomes. The goal of ESACC is to use the fewest nodes to achieve the best coverage. Therefore, in this study, the superiority of node scheduling is used as the adaptive function of ESACC. Using the perceptual coverage model described in Section 2.1, suppose the coverage vector of node $s_{i}$ is ${cov}_{i}=\left[\gamma_{i,1},\gamma_{i,2},\cdots ,\gamma_{i,G}\right]$, and the coverage vector of the other node $s_{j}$ is ${cov}_{j}=\left[\gamma_{j,1},\gamma_{j,2},\cdots ,\gamma_{j,G}\right]$, where i≠j. To determine whether an event is covered by a node, a Boolean operation is applied to the coverage vector by calculating the composite overlay vector of ${cov}_{i}$ and ${cov}_{j}$:

(9) $$\begin{array}{c} \Psi \left(s_{i},s_{j}\right)=\left[\gamma_{i,1}\vee \gamma_{j,1},\gamma_{i,2}\vee \gamma_{j,2},\cdots ,\gamma_{i,G}\vee \gamma_{j,G}\right] \end{array}$$

where Ψ represents the composite overlay vector, which is used to determine whether each event is covered by node $s_{i}$ and $s_{j}$. Therefore, the composite overlay vector of chromosome *k* can be expressed as

(10) $$\begin{array}{c} \Psi \left(k\right)=\left(A_{k1}\cdot {cov}_{1}\right)\vee \left(A_{k2}\cdot {cov}_{2}\right)\vee \cdots \vee \left(A_{kN}\cdot {cov}_{N}\right) \end{array}$$

Furthermore, the coverage of chromosome *k* can be expressed as

(11) $$\begin{array}{c} \lambda^{\left(k\right)}=\frac{{∥\Psi \left(k\right)∥}^{2}}{M} \end{array}$$

where ${∥\Psi \left(k\right)∥}^{2}$ represents the number of events covered by chromosome *k*. At this point, the utilization of the nodes in the network can be expressed as

(12) $$\begin{array}{c} \eta^{\left(k\right)}=\frac{\sum_{i=1}^{N}A_{k,i}}{N} \end{array}$$

where $\sum_{i=1}^{N}A_{k,i}$ indicates the number of nodes in the active state at the current time. The entire coverage-assessment process can be simplified to binary operations, greatly improving the performance of ESACC. From the aspect of evaluating the coverage of node-deployment algorithms, UASNs require the coverage of events to be as high as possible, and utilization of the nodes to be as low as possible. Therefore, we define an adaptive function $F^{k}$ to evaluate the advantages and disadvantages of chromosome *k*, and this is expressed as

(13) $$\begin{array}{c} F^{k}=\xi_{1}\cdot {\left(\lambda^{\left(k\right)}\right)}^{\alpha }-\xi_{2}\cdot {\left(\eta^{\left(k\right)}\right)}^{\beta } \end{array}$$

**Figure 3:**
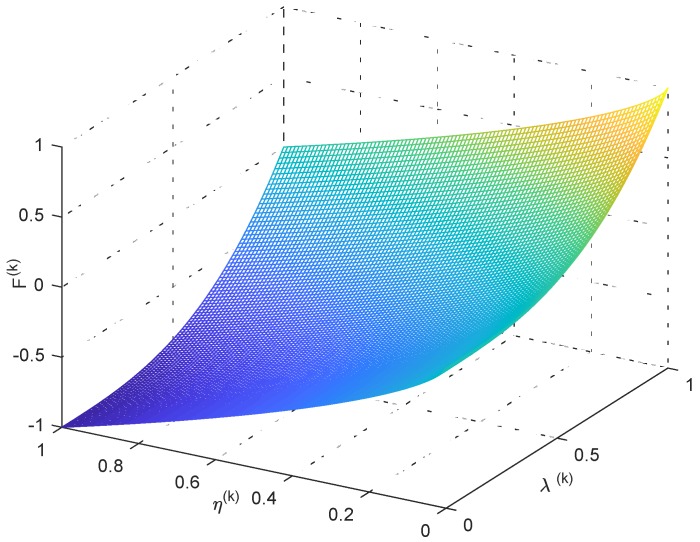
Relationship between values of $F^{k}$, α and β.

In the equation, $0\leq \lambda^{\left(k\right)}\leq 1$ and $0\leq \eta^{\left(k\right)}\leq 1$. $\xi_{1}$ and $\xi_{2}$ are the weight coefficients, whose values depend on the network designer’s comprehensive requirements for network performance indicators. α and β are exponential factors whose role is to distinguish the size of each chromosome $F^{k}$. The adaptive function $F^{k}$ is defined by Equation (13). It is a key factor affecting the performance of ESACC, and it includes four parameters: $\xi_{1}$, $\xi_{2}$ , α and β. Coverage rate $\lambda_{}^{\left(k\right)}$ and utilization $\eta_{}^{\left(k\right)}$ are equally important indicators for evaluating UASNs, so we set $\xi_{1}=\xi_{2}=1$. α = 3 and β = 0.7 is based on our practical experience and repeated experiments. Figure 3 shows the trend of the adaptive function $F^{k}$ when α = 3 and β = 0.7. The higher is the coverage rate $\lambda_{}^{\left(k\right)}$, the larger is the value of $F^{k}$, and the lower is the utilization rate of nodes $\eta_{}^{\left(k\right)}$, the larger is the value of $F^{k}$, which accord with the design intention.

### 3.3. Algorithm Simulation and Analysis

#### 3.3.1. Genetic Evolution

The selection operation selects good individuals from the current group, allowing them to breed the next generation as fathers. According to the above ESACC coding rules, the gene represents the status of the node: 1 indicates active, and 0 indicates sleep. The chromosomes with higher adaptability have better scheduling methods in the UASNs to handle the SCP problems, with a greater probability of being selected through the selection operation. This paper uses the roulette method, by which independent solutions are selected from a circle, and each individual’s probability of selection (reproduction probability) is proportional to its adaptability. The so-called reproduction probability is defined as

(14) $$\begin{array}{c} P_{i}={}^{f_{i}}\;\;∕\;\;{}_{\sum_{j=1}^{K}f_{j}}\; \end{array}$$

where $f_{i}$ is the adaptability of each individual $a_{i}$, and *k* is the population size. Interleaving in genetic algorithms means that new individuals can be created. In ESACC, each gene represents the state of a network node. In the scheduling algorithm of network nodes, the state changes of nodes are unrelated. Therefore, this paper selects the random-point crossover method to reproduce. Mutation applies only to offspring individuals that have been born from two parents. In a genome set composed of all offspring individuals, the genome is selected for mutation with mutation probability $P_{m}$. Mutation is an important flexible search operator, which can force the algorithm to search for new regions, thereby helping the genetic algorithm avoid premature convergence and resulting in a local optimal solution. In this paper, the mutation probability is the reciprocal of the length of the chromosome, which is theoretically proven to be optimal [27].

#### 3.3.2. Local Search

To further improve the adaptability of the genetic algorithm to calculate the population, we propose a simple local search strategy that makes ESACC converge quickly. For the next generation, this strategy determines whether to keep the modification by modifying the value of each gene in the chromosome (changing the value of a gene from 1 to 0) and comparing it with the adaptability of the original chromosome. This gives us a better node-scheduling scheme than the original node. Population *k* is expressed as $Pop_{k}=\left\{u_{1},u_{2},\cdots ,u_{k}\right\}$, where $u_{k}$ is the *k*th group of chromosomes, and $A_{i1},A_{i2},\cdots ,A_{iN}$ is the gene sequence of chromosome *i* , where $i\in \left[1,k\right]$ . The pseudo code of the local search strategy in ESACC is as follows in Algorithm 1.

**Algorithm 1** Local search algorithm.1: Calculate the adaptability $fit(A_{i})$ of chromosome $u_{i}$; 2: Statistics of index position *g* where gene $A_{ij}$ equals 1 in chromosome $u_{i}$, $j\in \left[1,N\right]$, $g\subset \left[1,N\right]$; 3: **for**
t=1,2, ⋯, length(g)
**do** 4:  **if**
$fit\left({A_{i}}^{\prime }\right)$ > $fit\left(A_{i}\right)$
**then** 5:   $fit\left(A_{i}\right)$ = $fit\left({A_{i}}^{\prime }\right)$; 6:   Keep the state of $A_{it}$; 7:  **else** 8:   $A_{it}$ = 1; 9:  **end if** 10: **end for** 11: Get the improved chromosome $u_{i}$ and repeat to obtain the optimal population $POP_{k}$.

### 3.4. Node Wake-Up Scheme

To achieve energy-saving coverage, we propose a node sleep-scheduling strategy based on a memetic algorithm. However, uncovered events will occur as certain nodes are gradually depleted of energy. Therefore, we propose a wake-up strategy to wake up some sleeping nodes for re-coverage. Assume that the power of the sink node of the network is sufficient to perform ESACC operations and wake-up schemes. Below, we describe the node wake-up scheme.

**Figure 4:**
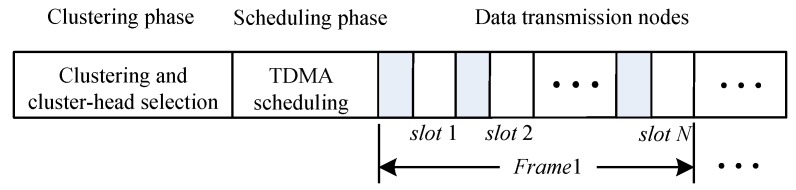
Time slot diagram of a TDMA frame period.

In UASNs, the node wake-up scheme runs in rounds, each of which includes clustering, scheduling, and data-transmission phases. In the clustering phase, nodes form several clusters through self-organizing and select one node in each cluster as the cluster head. The cluster head is responsible for forwarding messages received by the cluster node to the sink node. Then, it enters the scheduling phase. The UASNs applies a TDMA (Time Division Multiple Access) technique to accomplish communication in between the nodes and also their cluster head. Additionally, the cluster head has the ability to connect with the remote base station directly as well as accountable of forwarding the messages gotten from its cluster nodes to the base station. The head node will set up a TDMA timetable as well as send it to its cluster nodes. The cluster nodes could utilize the TDMA timetable to arrange the time slots for every node. Specifically, a TDMA frame consists of a fixed number of time slots, which equals the number of nodes in the cluster. The first time slot of each frame is reserved for the cluster head. After the inter-cluster communication link is established, it enters the data-transmission phase and the cluster members are designated to send data in their respective time slots. Compared with radio channels, the propagation delay of underwater channels is large. Therefore, the guard interval between slots should be set large enough to ensure that the cluster head has completed the reception of the current slot data before the data of the next slot arrives. In the entire network, the length of each frame period is uniform, which determines the interval between two consecutive transmissions. Since the guard interval may be long, the number of cluster members should not be excessive. Figure 4 shows a slot diagram of a TDMA frame period. After completing the data collection, it will enter the next round, re-elect a cluster head, and re-dispatch.

According to the node sleep-scheduling scheme, some nodes enter the sleep mode, and the rest are responsible for event monitoring. When the sink node receives a signal from a dead node, i.e., the energy of a certain node is about to run out, the wake-up scheme will wake some of the nodes in the next round. Assume that the energy of node $n_{i}$ is about to be exhausted ($E_{n_{i}}<E_{th}$). If node $n_{i}$ is active, then the original composite coverage vector of all nodes is $c_{n_{i}}^{\Delta }$. If node $n_{i}$ is dead, then the original composite coverage vector of all nodes is $c_{n_{i}}^{\nabla }$ . Then, according to the Boolean operation, the vector $c_{unc}^{n_{i}}$ that can be obtained by an uncovered event can be expressed as

(15) $$\begin{array}{c} c_{unc}^{n_{i}}=c_{n_{i}}^{\Delta }⊕c_{n_{i}}^{\nabla } \end{array}$$

where ⊕ denotes an exclusive-OR operation in Boolean operations; if optcom represents an optimal scheduling combination when node $n_{i}$ runs out, then the pseudo code of the node wake-up scheme is described as follows in Algorithm 2.

**Algorithm 2** Stud Crossover (SC) Operator1: **if**
$E_{n_{i}}<E_{th}$
**then** 2:  Find the neighbor node of $n_{i}$, $u_{i}$  $u_{i}=\left\{n_{j}\in C\left|d\left(n_{i},n_{j}\right)<r_{s},i\neq j,j\in \left[1,N\right]\right.\right\}$; 3:  Find ρ, which is the number of nonempty subsets Y of the neighbor node $u_{i}$  $Y=\left\{Y_{p}\left|\forall n^{*}\in Y_{p},\exists n^{*}\in u_{i}\right.\right\}$, $\rho =2^{length\left(u_{i}\right)}-1$; 4:  **for**
j=1,2, ⋯, ρ
**do** 5:   **for**
k=1,2,⋯, length($Y_{j}$) **do** 6:    $c_{\mathrm{cov}}^{Y_{k}}=c_{n\left[Y_{j}\left(k\right)\right]}⋃c_{\mathrm{cov}}^{Y_{k}}$; 7:   **end for** 8:   $Value=c_{\mathrm{cov}}^{Y_{k}}\;\&\;c_{unc}^{n_{i}}$; 9:   **if**
$\sum_{1\in Value}1>optval$
**then** 10:    optval = $Y_{j}$; 11:   **end if** 12:   **end for**   optcom; 13: **end if**


The main goal of the wake-up scheme is to generate an optimal scheduling combination to wake a node from sleep state by sending a wake-up signal to the neighbor node of the dead node. At the beginning of the next round, the sink wakes up some nodes according to the wake-up scheme. If a node dies again, then the wake-up scheme will reevaluate the coverage and decide to wake some other nodes to resume uncovered events.

### 3.5. Network Reconfiguration Mechanism

**Figure 5:**
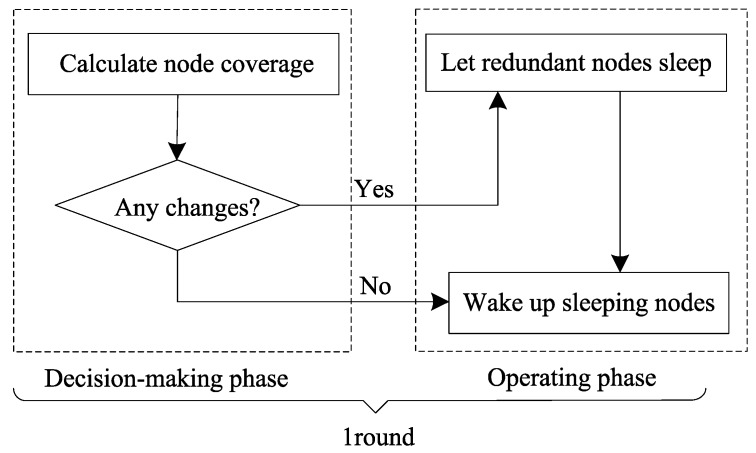
Network reconfiguration mechanism flow chart.

In actual situations, the events monitored by the sensor network may be relatively static or dynamic. For example, in the investigation of mineral resources on the seabed, events are stationary. Conversely, in marine water-quality monitoring, incidents to be monitored, i.e., pollutants, may drift along with the movement of seawater; in a dynamically changing situation, the sensor network and the events to be monitored are likely to be constantly changing. However, it costs too much for a node to update the event information every moment to perform intelligent processing or tracking. Therefore, it is necessary to reanalyze the node coverage after a certain period. We divide each round into decision-making and operational phases. The decision-making phase reevaluates the monitoring of events. In the operation phase, all nodes follow the ESACC plan according to the evaluation results in the decision-making phase until the end of the round. Figure 5 shows the flowchart of the network-reconfiguration mechanism.

## 4. Performance Evaluations

We evaluated the performance of the proposed ESACC scheme through extensive simulation tests. Assume a UASN contains preset numbers of nodes and events. Each node is static after a random or scheduled deployment, and the event may be affected by the flow of water. We verified the convergence of ESACC and display the experimental results.

### 4.1. Convergence of the ESACC

To prove the fast convergence characteristics of ESACC, under the same condition, we assessed the computational time and fitness of ESACC in UASNs of different sizes and compared it with the results of genetic algorithm (GA) [30]. We randomly deployed 64 events in 3D monitoring waters with a length, width and height of 100 m. The number of nodes deployed ranged between 50 and 500. No matter the number of sensor nodes deployed, the node’s perceived radius was 30 m. Every experiment was repeated 30 times.

In general, due to the evolutionary constraints of GA, it requires more generations to generate optimal solutions. However, with the use of additional local search schemes, ESACC is able to generate optimal solutions in fewer generations. Assume that both ESACC and GA use the same criteria to prevent evolution, and when the fitness of the best chromosome is above a predetermined threshold, the evolutionary process is terminated. Based on our previous experimental studies to determine fitness thresholds for different network sizes, we found that, based on experimental results, ESACC is highly adaptable around the second generation. Moreover, we also recorded the timestamp and fitness of the GA. Then, we compared the calculation time of ESACC with the calculation time of GA under different fitness values. The statistic results of experiments are as follows in Table 1.

It is worth noting that the computing platform is Intel (R) Core (TM) i7-3770 CPU @ 3.40 GHz, 8 GB memory. Table 1 plainly shows that the ESACC could offer an optimum schedule for nodes that is much better compared to that of GA. We could see that, with the same fitness, ESACC takes lower computing time to generate solutions with far better fitness compared to that in GA. As the network grows in size, the advantages of ESACC become more apparent. This is because the SCP becomes more complex as the number of nodes and events deployed increases. When it comes to network with 450 nodes, the computing time for the ESACC at a fitness value of 0.86 is 12.10 s, 81.6% faster than GA. In all other cases, the proposed ESACC is also able to achieve higher fitness and use less time. In summary, because the nodes deployed in the UASNs become more dense, the convergence and fitness generated by the ESACC is better than that in GA.

**Figure 6:**
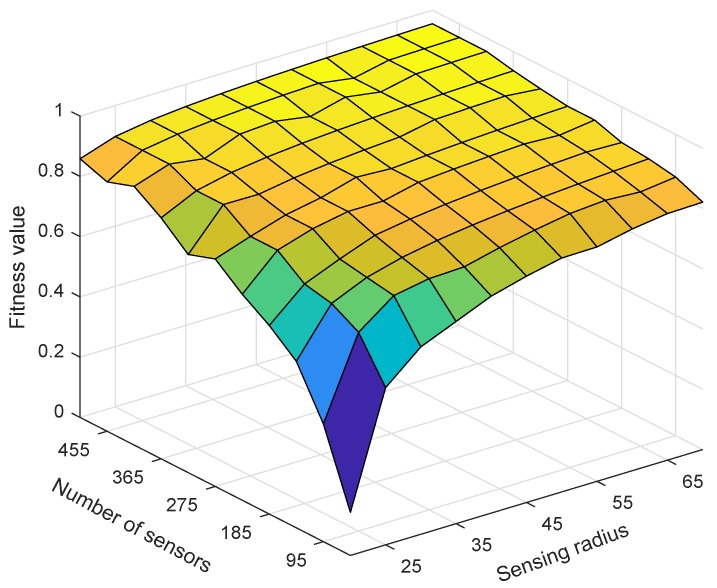
The change trend of the fitness function when the number of nodes changes and the node sensing radius $r_{s}$ changes.

**Figure 7:**
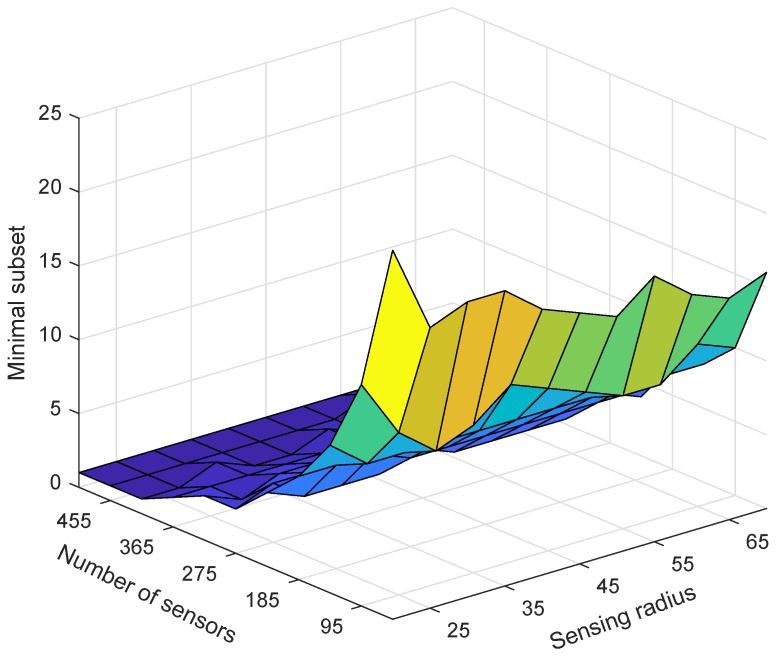
The change trend of the number of nodes in the minimum node set when the number of nodes changes and the node sensing radius changes.

Next, we tested the impact of changes in the number of nodes and sensing radius $r_{s}$ on ESACC performance. Figure 6 and Figure 7 show the effect of the sensing radius $r_{s}$ from 20 m to 70 m and the number of nodes from 50 to 500 on ESACC performance (fitness value and minimum number of node subsets) when nodes are randomly distributed in the 3D water environment monitoring area. It can be seen in Figure 6 that, as the sensing radius $r_{s}$ or the number of nodes increases, the fitness value increases, which is mainly because, as the sensing radius $r_{s}$ or the number of nodes increases, the union of the sensing ranges of nodes in the network increases. As a result, the network coverage is also increased. From another perspective, the number of redundant nodes in the network also increases, and the number of nodes that need to sleep will become more and more, which will reduce the node utilization. This shows that the fitness function proposed in this paper can effectively evaluate network performance.

Figure 7 depicts the change trend of the number of nodes in the minimum set of nodes as the number of nodes changes and the sensing radius $r_{s}$ of the nodes changes. It is easy to be seen that, as the sensing radius $r_{s}$ or the number of nodes increases, the number of nodes in the minimum set of nodes decreases. This is mainly because, as the sensing radius $r_{s}$ or the number of nodes increases, the number of redundant nodes in the network increases, and the number of nodes that need to sleep also increases. In summary, experiments show that ESACC can effectively reduce the number of active nodes and reduce node utilization based on network status.

### 4.2. Algorithm Simulation and Analysis

We used MATLAB for simulation. To eliminate the randomness of the experiment, the final result was taken as the average of 30 experiments. Assuming that the three-dimensional monitoring water area is 200 m in each dimension, the sink node is located at the center of the water surface, and 64 events are to be monitored. Each node has the same initial energy, and all nodes are assumed to be homogeneous. Table 2 shows the meanings and values of simulation parameters.

We now demonstrate ESACC’s performance evaluation results in extending the life cycle and maintaining coverage of UASNs. ESACC was applied to the sink node. By controlling the sleep or active state of the node, the node energy can be fully utilized while maintaining efficient coverage of the event. Assume that the sink node has enough power to schedule every node in the network. When a node’s energy is below a critical value, the ESACC wake-up scheme can determine which neighbor must be awakened to recover uncovered events.Therefore, the network coverage, average remaining energy of network nodes, and network runtime need to be considered simultaneously.

To compare the performance of the ESACC with those of LEACH-Coverage-U [11] and CMSS algorithm [6] (the CMSS algorithm is a typical algorithm for extending the network lifetime through sleeping scheduling), the network parameter settings mentioned above were used for simulation. It should be noted that all experimental designs here are based on the idea of clustering.

**Figure 8:**
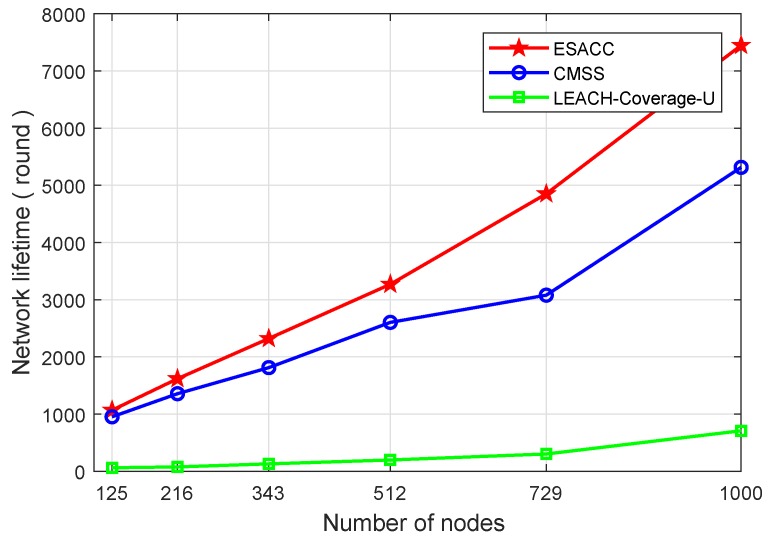
Relationship between network lifetime and number of nodes.

**Figure 9:**
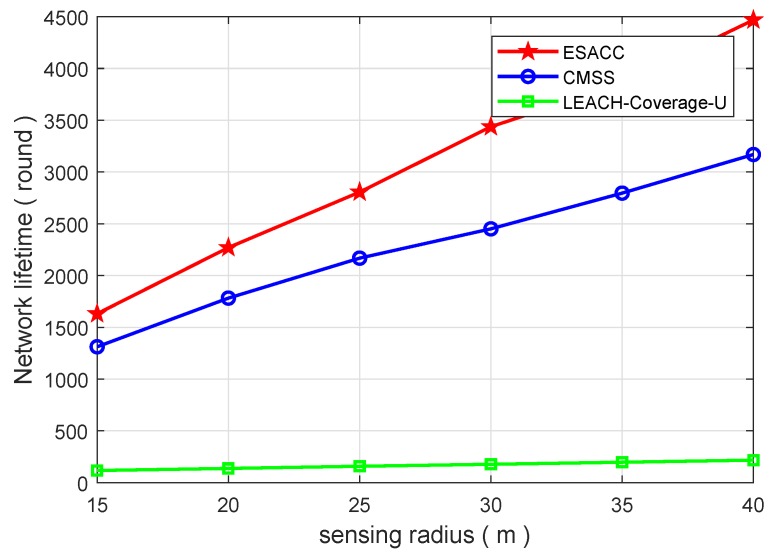
Relationship between network lifetime and sensing radius $r_{s}$.

Figure 8 and Figure 9 show the comparison of the network lifetimes of the above three algorithms. Figure 8 is a comparison of the network lifetime of the ESACC algorithm with the CMSS and the LEACH-Coverage-U algorithm when the number of events is fixed at 64, the node sensing radius $r_{s}$ is fixed at 20 m, and the number of nodes varies from 125 to 1000. As can be seen in the figure, in terms of average network lifetime, the ESACC algorithm is 951.8∼2028.9% better than EACH-Coverage-U and 12.2∼57.6% better than CMSS. Figure 9 is a comparison of the network lifetime of the ESACC algorithm with the CMSS and LEACH-Coverage-U algorithm when the number of events is fixed at 64, the number of nodes is fixed at 343 , and the node sensing radius $r_{s}$ varies from 15 to 40. It is easy to find that the proposed ESACC algorithm is 1294.1∼1958.9% better than EACH-Coverage-U, and 24.3∼40.9% better than CMSS. Obviously, the proposed ESACC algorithm shows good performance compared to other existing methods.

**Figure 10:**
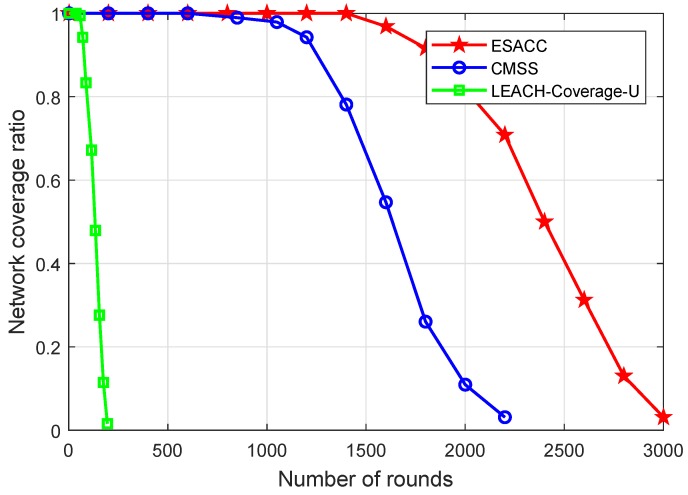
Relationship between network coverage and running rounds.

Figure 10 is a comparison of the network coverage of the ESACC algorithm and the CMSS and LEACH-Coverage-U algorithms with the change of rounds in the network when the number of nodes is fixed at 512, the node sensing radius $r_{s}$ 20 m, and the event 64. It can be seen that, when the number of running rounds is the same, the network coverage rate of the LEACH-Coverage-U algorithm is significantly lower than that of ESACC and CMSS. Because it does not apply a node scheduling strategy, this leads to a large number of nodes dying prematurely, which in turn leads to an increase in network vulnerabilities. CMSS and ESACC not only consider network coverage, but also consider the sleep and wake-up mechanisms of redundant nodes. Compared to the CMSS, ESACC can maintain 97% high coverage over a long period of time (from the initial time until 1327 rounds) in its lifetime (3269 rounds). This is because the ESACC algorithm improves the efficiency of network scheduling and reduces the energy loss caused by frequent communication between nodes in the network, so that more nodes with sufficient energy participate in network monitoring tasks, thereby improving network service quality.

**Figure 11:**
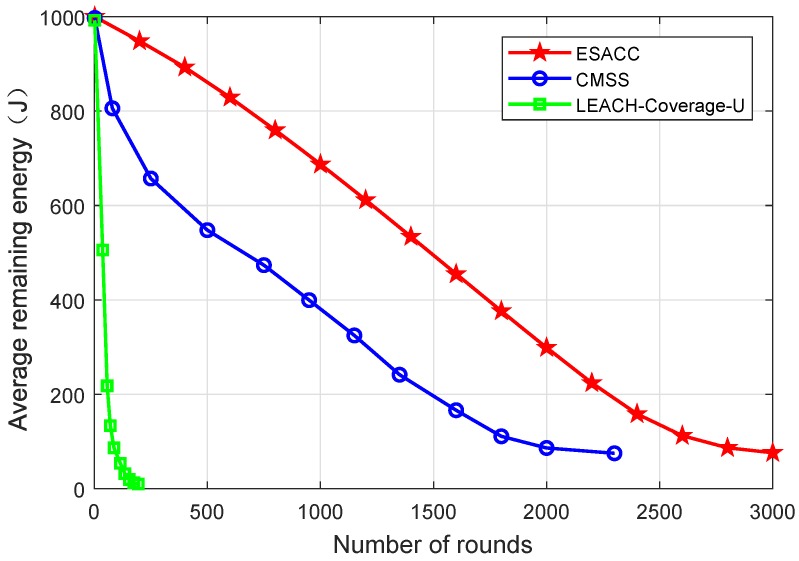
Average residual energy of network nodes.

Figure 11 is a comparison of the average remaining energy of the network nodes of the ESACC algorithm with CMSS and LEACH-Coverage-U with the change of rounds in the network when the number of nodes fixed at 512, the node sensing radius 20 m, and the number of events 64. It can be seen that, when the number of running rounds is the same, compared with the LEACH-Coverage-U algorithm, the network nodes of CMSS and ESACC have a lower energy gradient and a higher energy utilization rate. This is mainly because the CMSS and ESACC algorithms optimize the network structure, close redundant nodes, and reduce the overall energy consumption of the network. At the same time, ESACC shows better energy utilization than CMSS. This is because, when a dead node appears in the network, based on the minimum set of nodes, the ESACC algorithm seeks the optimal combination by waking up the neighbor nodes of the dead node, making up for the lack of node death, and making the energy consumption of the network balanced, thereby extending the network lifetime.

## 5. Conclusions

A UASN is different from a wireless sensor network on land. It must account for the particularity of the underwater acoustic environment, and it requires greater energy efficiency. Therefore, this paper proposes an energy-saving coverage-scheduling algorithm called ESACC. A memetic algorithm is used to implement the sleep scheduling of redundant nodes, which simplifies the network topology and reduces the network energy consumption. A wake-up scheme is also established. The use of some dormant redundant nodes in the network to replace nodes that are about to die due to energy exhaustion maintains high network coverage. The algorithm uses a sleep–wake strategy to reduce network energy consumption and extend the network life cycle while maintaining high coverage. Simulation results show that, compared with the CMSS and LEACH-Coverage-U algorithms, ESACC can close redundant nodes and maintain UASN operation with the minimum number of active nodes. When a node’s energy is close to critical, it can wake up a sleeping node to maintain high coverage. Next, applying ESACC to energy-saving deployment in UASNs is an important aim of future work.

## Figures and Tables

**Table 1:** The statistic results of experiments. Each experiment was repeated 30 times to compare the average computation time (seconds) between ESACC and GA at different fitness values.

Number	Fitness	ESACC	Std.	GA	Std.	Number	Fitness	ESACC	Std.	GA	Std.
50	0.62	0.35	0.08	0.09	0.02	150	0.79	3.03	0.07	8.53	3.54
50	0.58	0.26	0.07	0.07	0.02	150	0.75	1.49	0.05	4.28	2.24
50	0.37	0.01	0.01	0.01	0.01	150	0.36	0.03	0.01	0.05	0.01
250	0.82	4.7	0.11	31.16	16.52	350	0.85	8.91	0.21	52.64	25.36
250	0.81	3.91	0.11	18.56	10.23	350	0.83	7.37	0.20	40.52	21.23
250	0.35	0.05	0.01	0.09	0.01	350	0.34	0.07	0.01	0.12	0.02
450	0.87	16.61	0.28	74.52	36.14	550	0.88	20.07	0.21	118.11	55.21
450	0.86	12.10	0.27	68.48	33.74	550	0.87	18.01	0.30	100.11	52.62
450	0.34	0.09	0.01	0.15	0.01	550	0.34	0.12	0.01	0.19	0.02

**Table 2:** Simulation scenarios and parameters.

Parameter	Value	Parameter	Value
Initial energy of node $E_{O}$	1000 J	Node energy threshold $E_{th}$	10 J
Carrier frequency *f*	25 kHz	Packet size $M^{\prime }$	1 kbit
Node sensing radius $R_{s}$	30 m	Circuit energy consumption to process one bit of information $E_{elec}$	50 nJ/bit
Average depth *H*	100 m	Data transmission rate $S_{v}$	5 kbps

## References

[1] Pompili D., Akyildiz I.F. (2009). Overview of networking protocols for underwater wireless communications. IEEE Commun. Mag..

[2] Zheng P. (2012). Effcient and Robust Underwater Sensor Network Systems: Design, Implementation and Experiments. Ph.D. Dissertation.

[3] Akyildiz I.F., Pompili D., Melodia T. (2005). Underwater acoustic sensor networks: Research challenges. Ad Hoc Netw..

[4] Cui J.H., Kong J., Gerla M., Zhou S. (2006). The challenges of building mobile underwater wireless networks for aquatic applications. IEEE Netw..

[5] Liu F., Tsui C.Y., Zhang Y.J. (2010). Joint Routing and Sleep Scheduling for Lifetime Maximization of Wireless Sensor Networks. IEEE Trans. Wirel. Commun..

[6] Danratchadakorn C., Pornavalai C. Coverage maximization with sleep scheduling for wireless sensor networks. Proceedings of the IEEE International Conference on Electrical Engineering/electronics, Computer, Telecommunications and Information Technology.

[7] Zhao Y., Wu J., Li F., Lu S. (2012). On Maximizing the Lifetime of Wireless Sensor Networks Using Virtual Backbone Scheduling. IEEE Trans. Parallel Distrib. Syst..

[8] Lin C.C., Deng D.J., Wang S.B. (2016). Extending the Lifetime of Dynamic Underwater Acoustic Sensor Networks Using Multi-Population Harmony Search Algorithm. IEEE Sens. J..

[9] Wang C., Lin H., Jiang H. (2016). CANS: A Congestion-Adaptive WSN-Assisted Emergency Navigation Algorithm with Small Stretch. IEEE Trans. Mob. Comput..

[10] Fan X., Chen Q., Che Z., Hao X. (2017). Energy-Efficient Probabilistic Barrier Construction in Directional Sensor Networks. IEEE Sens. J..

[11] Tsai Y. (2007). Coverage-preserving routing protocols for randomly distributed wireless sensor networks. IEEE Trans. Wirel. Commun..

[12] Ogundile O.O., Alfa A.S. (2017). A Survey on an Energy-Efficient and Energy-Balanced Routing Protocol for Wireless Sensor Networks. Sensors.

[13] Fariborzi H., Moghavvemi M. (2009). EAMTR: Energy aware multi-tree routing for wireless sensor networks. IET Commun..

[14] Zhang R., Labrador M.A. Energy-aware topology control in heterogeneous wireless multi-hop networks. Proceedings of the International Symposium on Wireless Pervasive Computing.

[15] Kar K., Kodialam M., Lakshman T.V., Tassiulas L. Routing for network capacity maximization in energy-constrained ad hoc networks. Proceedings of the 22th Annual Joint Conference of the IEEE Computer and Communications Societies.

[16] Zhang B., Zhao Z., Ma J., Mouftah H.T. Efficient Localized Topology Control Algorithm. Proceedings of the 2007 Workshop on High PERFORMANCE Switching & Routing.

[17] Jiang P., Liu J., Feng W., Wang J., Xue A. (2016). Node Deployment Algorithm for Underwater Sensor Networks Based on Connected Dominating Set. Sensors.

[18] Sozer E.M., Stojanovic M., Proakis J.G. (2000). Underwater acoustic networks. IEEE J. Ocean. Eng..

[19] Zhao L., Liang Q. (2006). Optimum cluster size for underwater acoustic sensor networks. Proceedings of the IEEE Conference on Military Communications.

[20] Jiang P., Liu J., Wu F. (2015). Node non-uniform deployment based on clustering algorithm for underwater sensor networks. Sensors.

[21] Stojanovic M. (2007). On the Relationship Between Capacity and Distance in an Underwater Acoustic Channel. ACM Sigmob. Mob. Comput. Commun. Rev..

[22] Berkhovskikh L., Lysanov Y. (1982). Fundamentals of Ocean Acoustics.

[23] Ganesan T., Elamvazuthi I., Shaari K.Z.K., Vasant P. (2013). Swarm intelligence and gravitational search algorithm for multi-objective optimization of synthesis gas production. Appl. Energy.

[24] Ting C.K., Liao C.C. (2010). A memetic algorithm for extending wireless sensor network lifetime. Inf. Sci..

[25] Neshat M., Sepidnam G., Sargolzaei M., Toosi A.N. (2014). Artificial fish swarm algorithm: A survey of the state-of-the-art, hybridization, combinatorial and indicative applications. Artif. Intell. Rev..

[26] Du H., Na X., Rong Z. (2014). Particle Swarm Inspired Underwater Sensor Self-Deployment. Sensors.

[27] Liao C.C., Ting C.K. (2018). A Novel Integer-Coded Memetic Algorithm for the Set k-Cover Problem in Wireless Sensor Networks. IEEE Trans. Cybern..

[28] Moscato P. (1989). On Evolution, Search, Optimization, Genetic Algorithms and Martial Arts: Towards Memetic Algorithms.

[29] Trivedi A., Srinivasan D., Sanyal K., Ghosh A. (2017). A Survey of Multiobjective Evolutionary Algorithms Based on Decomposition. IEEE Trans. Evolut. Comput..

[30] Zhang Y., Zhou Z., Zhao D., Sun Y., Xue X., Ma L., Khreishah A., Zhang Y., Yan M. (2017). A Genetic Algorithm Based Mechanism for Scheduling Mobile Sensors in Hybrid WSNs Applications. Wireless Algorithms, Systems, and Applications (WASA 2017).

